# Two new mountainous species of *Lactuca* (Cichorieae, Asteraceae) from Iran, one presenting a new, possibly myrmecochorous achene variant

**DOI:** 10.3897/phytokeys.11.2563

**Published:** 2012-04-18

**Authors:** Norbert Kilian, Seyyedeh Bahereh Djavadi, Majid Eskandari

**Affiliations:** 1 Botanic Garden and Botanical Museum Berlin-Dahlem, Dahlem Center of Plant Sciences, Free University Berlin, Königin-Luise-Str. 6–8, 14195 Berlin, Germany; 2 Department of Botany, Iranian Research Institute of Plant Protection, P.O. Box 1454, Tehran 19395, Iran

**Keywords:** Compositae, Asteraceae, Cichorieae, *Lactuca rosularis* group, *Lactuca denaensis*, *Lactuca hazaranensis*, taxonomy, Iran, Kerman, Hazaran Mts, Zagros Mts, carpology, dissemination, elaiosome, myrmecochory

## Abstract

It is shown that the concept of the Iranian endemic *Lactuca polyclada* in the sense of both its original author Boissier and its current use actually admixes two entirely different species, as was first noted by Beauverd a hundred years ago but has been neglected by later workers. One is a putative relative of *Lactuca rosularis*, the other was recognised by Beauverd as a member of the genus *Cicerbita*. The name *Lactuca polyclada* Boiss. is lectotypified here, maintaining its use as established by Beauverd for the *Cicerbita* species. Both species are morphologically delimited and mature achenes of *Cicerbita polyclada* are illustrated for the first time. The putative relative of *Lactuca rosularis*, a rare local endemic of the summit area of Kuh e-Dena, which has remained without a valid name by now, is described as a new species, *Lactuca denaensis* N. Kilian & Djavadi, and illustrated. A third member of the *Lactuca rosularis* group, *Lactuca hazaranensis* Djavadi & N. Kilian, discovered among a recent collection and apparently being a rare chasmophyte of the Hazaran mountain massif in the province of Kerman, Iran, is described as a species new to science, illustrated and delimited from the other two species. This new species has peculiar achenes representing a hitherto unknown variant: the body of the beaked achenes is divided into two segments by a transversal constriction in the distal third. The proximal segment contains the embryo, the distal segment is solid with a lipid-containing yellow tissue. The easily detachable pappus and the equally easily detachable beak potentially obstruct dispersal by wind. Since detachment of the beak also exposes the lipid-containing tissue of the distal segment, its potential as an elaiosome and myrmecochory as a possible mode of dispersal are discussed.

## Introduction

Identification of a collection of a Cichorieae species, made by the third author together with A. Torabi in August 2010, in Kerman Province in the vicinity of the famous waterfalls near the city of Rayen on the eastern foot of Mt Hezar, revealed that it apparently represents a hitherto unknown lactucoid species. The species has strikingly unusual achenes, which show a transversal constriction in the distal third. Morphological comparison and evaluation of its affinities led to the discovery of another still unnamed species among the putatively related species.

The lactucoid genera, which form the subtribe Lactucinae, have a worldwide distribution in the northern hemisphere but extend also into the southern hemisphere in Africa and comprise about 230 species (Kilian et al. 2009). Iran belongs to the regions with a higher diversity of Lactucinae species. In the Flora Iranica area, seven lactucoid genera (*Cephalorrhynchus*, *Cicerbita*, *Lactuca*, *Mulgedium*, *Prenanthes*, *Scariola*, *Stepto-rhamphus*) with altogether c. 36 species, are known to science (Tuisl 1977), of which 22 occur in Iran itself. Relationships and delimitation of the lactucoid genera have been disputed since the time of Linnaeus and their circumscription is not yet settled. Ongoing morphological-molecular studies (Kilian et al. in prep.) reveal a considerable extent of homoplasy in morphological features, explaining the tremendous difficulties which all morphological attempts aiming at a natural classification of the lactucoid taxa have faced.

This paper (a) gives the description and delimitation of hitherto unknown or neglected plants, respectively, as two species new to science, (b) clarifies their morphological affinities among the lactucoid species, and (c) considers the peculiar achene morphology found in one of the two species with respect to its possible function for fruit dispersal.

## Material and methods

The study is based on herbarium material of the Herbarium of the Iranian Research Institute of Plant Protection (IRAN) and of the Herbarium of the Botanic Garden and Botanical Museum Berlin-Dahlem (B) as well as on digitised type material from the herbaria of G, M, MO, P, WAG (herbarium abbreviations according to Thiers 2008+). Digitised specimens were received upon request from the herbaria, viewed via the online herbarium catalogues of the herbaria or via JSTOR (2012), respectively.

The authors observed all morphological data presented and used in the description and comparison of the new species on the herbarium material cited in the text under the new species or in the Appendix, respectively. Micromorphological features were examined under a WILD M5 optical reflected-light microscope. Documentation of morphological features was done with an Olympus DP72 digital camera mounted on an Olympus SZX16 stereo zoom optical reflected-light microscope equipped with the Olympus analySIS docu software.

Light microscopic histochemical analysis of the achene tissue to test the presence of lipids was performed using Sudan III staining following Wanner (2004: 21, 24).

## Results and discussion

### Lactuca denaensis

N. Kilian & Djavadi sp. nov.

urn:lsid:ipni.org:names:77118676-1

http://species-id.net/wiki/Lactuca_denaensis

**Figure 1.**
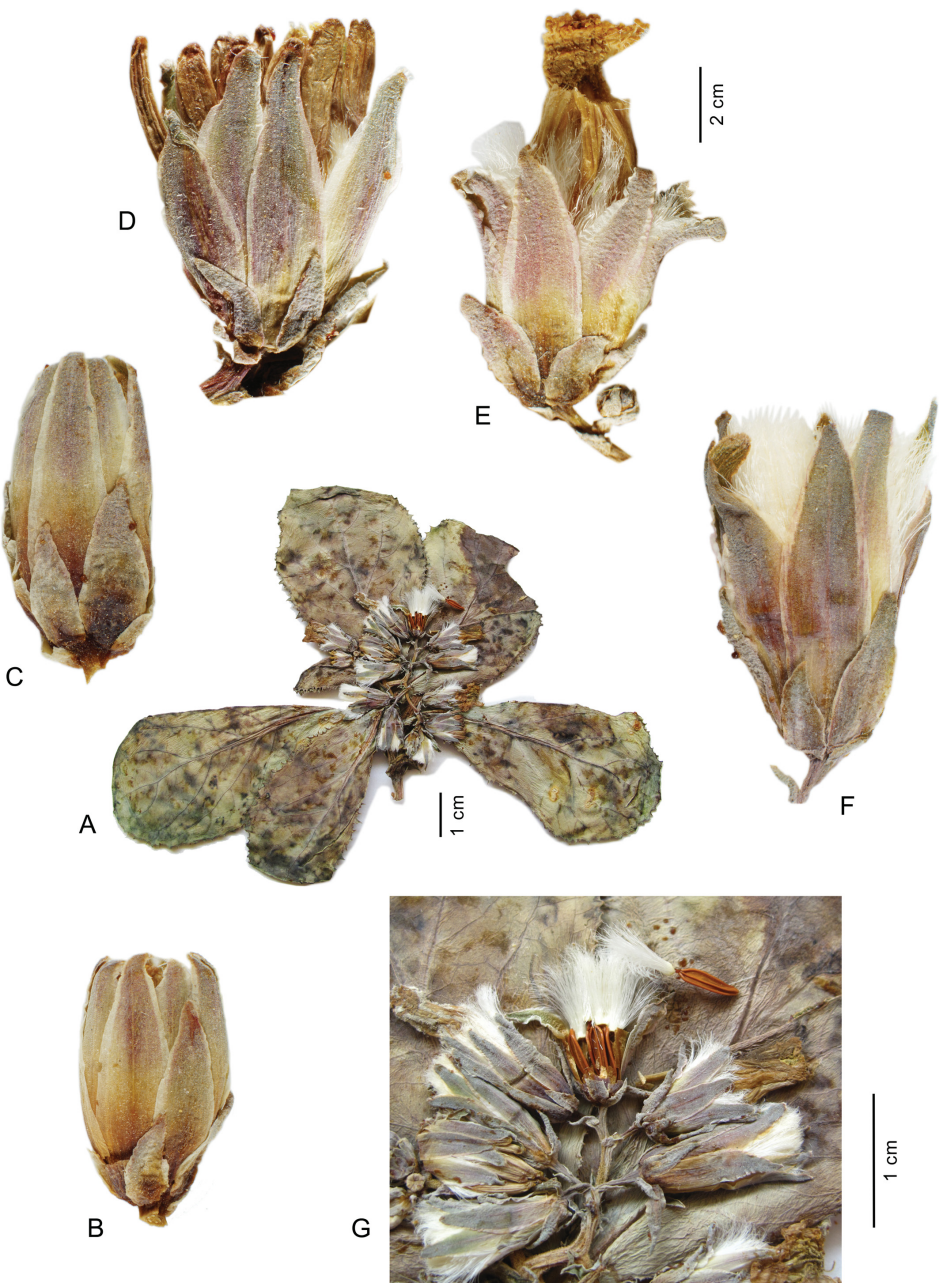
*Lactuca denaensis* – **A** habit, acaulescent form **B–F** capitula, all to the same scale, before flowering (**B–D**), with faded flowers (**E**), after flowering (**F**); **G** details of synflorescence, one capitulum at fruiting. – All from Remaudière, 5252E (IRAN 10625)

Fig. 1 3C; Tuisl 1968: t. 2, fig. 12–13 = Tuisl 1977: t. 201, fig. 9–10 under Lactuca polyclada (achene, aspect and cross section)

Lactuca polyclada sensu Boissier (1846: 10, 1875: 812) p.p. et Tuisl (1968: 606, 1977: 190–191) p.p., non sensu lectotypi.

#### Diagnosis.

Habitually similar to *Lactuca rosularis* but clearly distinguished by the rosette leaves being undivided (instead of lyrately to irregularly pinnatifid to pinnatisect), the involucre being 10–12 mm (instead of 6–9(–10) mm) long, the achenes having a 4–5 mm (instead of 2.2–3 mm) long corpus, a 0.4–0.9(–2) mm (instead of (1.1–)3–5.3 mm) long beak and a 5–6 mm (instead of 2.5–3 mm) long pappus.

#### Holotype.

[Iran, Kogiluyeh & Boyer Ahmad], in glareosis alpis Kuh-Daëna, fl. lutescens, 14 Jul 1842, Kotschy Pl. Pers. autral. 662 (G-BOIS G00330211, specimen annotated by Beauverd in 1910, see CHG 2012); selected isotypes: B 100426936, M 0030847 [p.p., two rosulate plants on the left], G [3 sheets], MO 6264530 [p.p., rosulate plants on the left and right], P 00750254 [p.p., left plant], P 00750251 [p.p., second bottom right rosulate plant], P 00750252 [p.p., first bottom right rosulate plant], P 00750253 [p.p., three rosulate plants at the bottom], WAG 0004075 [p.p., sterile leaf rosette on the left].

#### Description. 

Perennial rosulate herb, with a woody subterranean caudex, rosette shoots often on vertical subterranean axes vested with cataphylls below the sometimes somewhat elongate rosette, acaulescent to usually very shortly caulescent and less than 5 cm tall (Fig. 1A), rarely to c. 15 cm. Stem one per leaf rosette, usually not or little projecting above the leaf rosette. Rosette leaves (Fig. 1A) obovate to spatulate, tapering towards base, 2–7 × (1–)1.5–3.2 cm, somewhat glaucous; base semiamplexicaul, margin ± densely dentate and denticulate, apex rounded to, more rarely, subacute. Synflorescence corymbosely paniculiform, condensed through conspicuously short axes, of c. 6–20 capitula (Fig. 1A, G); peduncles 0.4–0.7 cm long. Capitula with c. 10–14 flowers. Involucre (Fig. 1B–F) narrowly cylindric at anthesis, 10–12 mm long, not elongating during maturation; outer phyllaries imbricate, outermost ± narrowly ovate, c. 2 mm long, following ones gradually longer, lanceolate, the longest up to c. 1/2 as long as inner ones; inner phyllaries linear-lanceolate, 7–8, ± in one row, subequal in length, with ± narrow scarious margin. Receptacle flat to slightly convex, naked. Flowers with corolla yellow, ligule 6.7–7.5 mm, tube 5–5.5 mm long; anther tube without appendages 3.2–3.5 mm, basal appendages c. 0.4–0.6 mm, apical appendages 0.3–0.4 mm long; style arms 2.8–3.2 mm long. Achenes (Fig. 1G, 3C) homomorphic, including beak 4.8–5.7 mm long; corpus 4–5 mm long, up to 1.1–1.3 mm in diam., slender-obovoid, compressed, apex contracted into a stout, easily detachable beak c. 0.4–0.9(–2) mm long; achene body apart from the two lateral ribs with 1 similarly strong median rib on either side, rarely dorsally with 2 equally strong ribs, secondary ribs missing; achene surface ± smooth, brown. Pappus simple, without an outer series of minute hairs, setae thin, white, 5–6 mm long, persistent. – Flowering and fruiting: July to September.

#### Notes.

Boissier (1846) described a new species, *Lactuca polyclada*, based on two collections (no. 603 and 662) made by T. Kotschy in the Zagros mountains, in the first half of July 1842. Coming from the village Dozdkurd (Edmondson and Lack 2006), Kotschy collected the material on the upper slopes of the “Kuh Daena”[= Kuh e-Dena, c. 30°56'N, 51°28'E; summit c. 4448 m, see Wikipedia (2012), situated in the present-day province Kogiluyeh & Boyer Ahmad of Iran]. According to Kotschy’s original labels present on the sheets in the Boissier herbarium in Genève (G-BOIS), the collection Kotschy 603 was collected on 10 July and Kotschy 662 on 14 July 1842. The entire material of Kotschy’s gatherings from S Iran was revised by Boissier and subsequently edited and distributed by R. Hohenacker in his series of exsiccatae “Plantarum Persiae australis siccatarum species 440, collectae a Th. Kotschy, determinatae a Dre E. Boissier, editae a R. F. Hohenacker” (Triebel and Scholz 2001+; Edmondson and Lack 2006). Duplicates are present today in many herbaria. The material of *Lactuca polyclada* was distributed as a single item under the united numbers “603. 662.” and with a single collecting date cited as “14 Jul 1842” on the printed label. The syntypes in Boissier’s herbarium as well as the duplicates distributed by Hohenacker in this series of exsiccatae as no. “603. 622.” contain two morphologically distinct elements: (a) small leaf rosettes with usually very short, branched, slender to capillaceous flowering axes; (b) almost leafless, from base on divaricately and intricately branched, conspicuously inflated flowering axes. Apparently it has been taken as evident by Boissier and later workers that both elements represent different forms or stages of development of the same species. This assumption is backed by the leaves, which are fairly similar in colour, size, shape and denticulation of the margin in both elements, as well as by the existence of a plant with exceptionally well developed flowering shoots, approaching those of Kotschy 603, among the material of Kotschy 662 (on G00330211, the holotype sheet of *Lactuca denaensis*). Boissier (1846: 10) expressed this hypothesis in the following way: “panicula corymbosa intra folia subsessili ... Post anthesin saepe panicula valde augetur, ramosissima fit semipedalis ramis elongatis intricatis dichotomis spongiose incrassatis, sed haec forma monstrosa est, nam in ea nunquam achenia perfecta observavi.” [“with a corymbose panicle subsessile among the leaves ... after anthesis panicle often strongly enlarged, very much branched, becomes half a foot long, with elongate, intricate, dichotomous, softly inflated branches, but this is a monstrous form, because I never have observed a perfect achene in it.”]. Tuisl (1977: 191) considered the two elements as different developmental stages: “caulis florifer abbreviatus, fructifer 10–30 cm longus” [“stems at anthesis very short, in fruit 10–30 cm long”].

**Figure 2.**
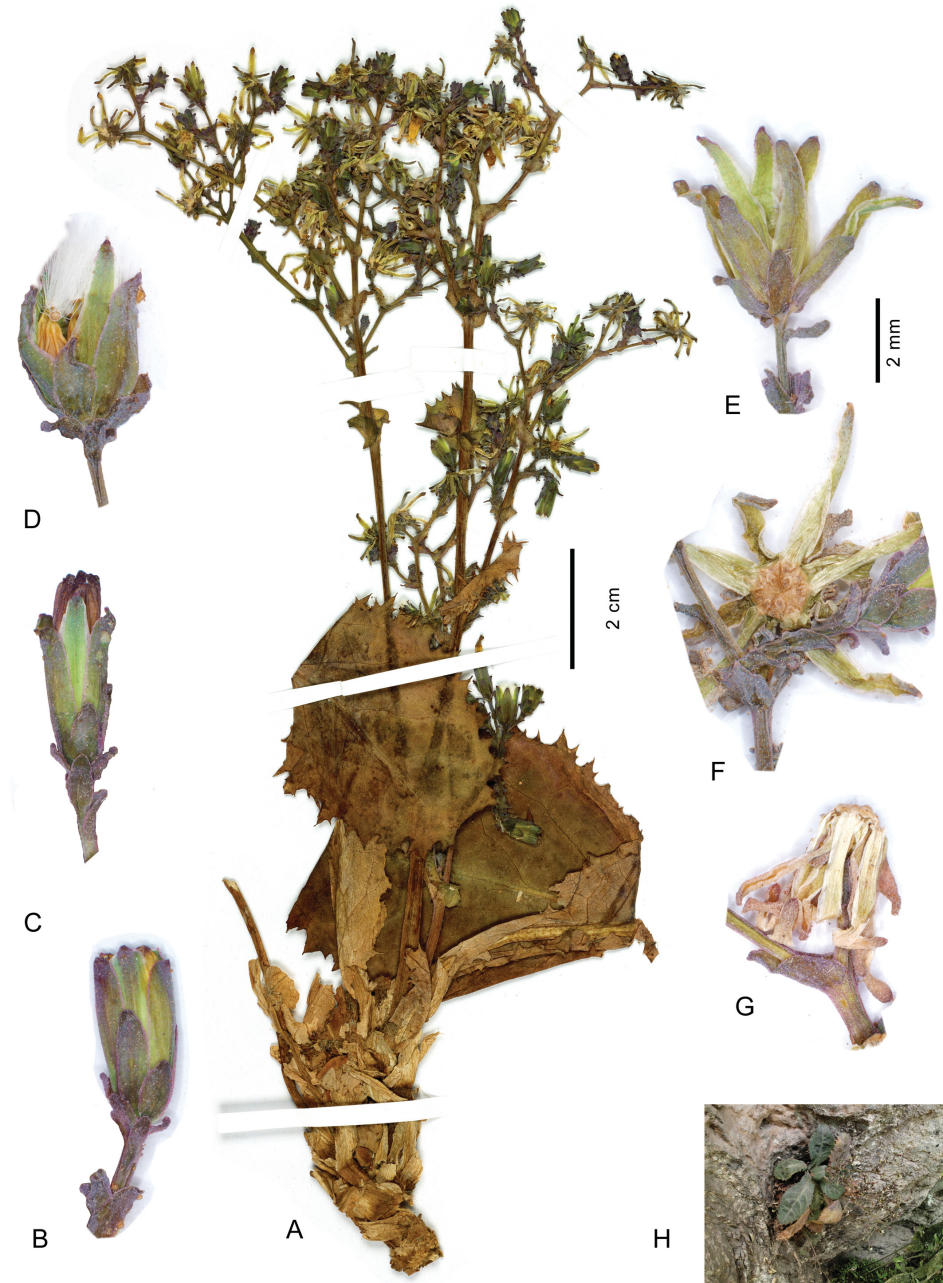
*Lactuca hazaranensis* – **A** habit, left plant of holotype sheet **B–G** capitula, all to the same scale, before flowering (**B–C**), at fruiting (**D–E**), after fruiting (**F–G**); **H** plant in natural habitat **A–G** from the holotype **H** plant in the natural habitat at the type locality; photograph by M. Eskandari, 10 Aug 2010.

Beauverd (1910: 131–132), in contrast, came to the conclusion that the syntypes of *Lactuca polycalda* in the herbarium of Boissier (G-BOIS) represent two entirely different species and correctly distinguished them. Beauverd (1910: 131) consequently restricted the name *Lactuca polyclada* to “K[otsch]y 603 solum! excl. No. 662 et descr. achen.,” and formed the new combination *Cicerbita polyclada* (Boiss.) Beauverd for the taxon with divaricately branched inflated stems, smaller involucres, bluish flowers and a pappus with an outer row of minute hairs. Mature fruits of *Cicerbita polyclada*, missing in Kotschy 603, are known through a collection of T. Strauss (at B, see Appendix). They are illustrated here for the first time and show further differences between the two species, in particular with respect to the prominence of the ribs and shape and structure of the body apex (compare Fig. 2C and D–E). For a summary of the differences see Table 1.

According to Art. 7.11 of the Vienna Code, Beauverd’s restriction of the name to Kotschy 603 does not, however, constitute a lectotypification, because he did not use “the term ‘type’ (typus) or an equivalent” (McNeill et al. 2006). Later authors apparently neglected Beauverd’s rectification and we are also not aware of any lectotypification of Boissier’s name. In accordance with Art. 9.12 (McNeill et al. 2006) and in this way maintaining the only unequivocal use of the name as established by Beauverd (1910: 131) for the species placed by him in *Cicerbita*, we formally lectotypify *Lactuca polyclada* Boiss. following his restriction: *Cicerbita polyclada* (Boiss.) Beauverd in Bull. Soc. Bot. Genève 2: 131. 1910 ≡ *Lactuca polyclada* Boiss., Diagn. Pl. Orient., ser. 1, 7: 10. 1846. – Lectotype (designated here): [Iran, Kogiluyeh & Boyer Ahmad], in glareosis ad latera septentr. m. Kuh-Daëna, flos caeruleus, 10 Jul 1842, Kotschy Pl. Pers. autral. 603 (G-BOIS G00330212 [2 sheets annotated by Beauverd in 1910], see CHG 2012).

The second species admixed by Boissier under the name *Lactuca polyclada* with a usually very short or almost missing flowering stem, yellow flowers, a pappus without an outer row of minute hairs and the achene anatomy as illustrated by Tuisl (1968: t. 2, fig. 12–13 = 1977: t. 201, fig. 9–10) was left by Beauverd without a legitimate name. We have named and described it therefore here as *Lactuca denaensis*, the name being typified with the syntype “Kotschy 662” of *Lactuca polyclada* Boiss. in Boissier’s herbarium. Since Kotschy’s both collections no. 603 and 662 were combined by Hohenacker in his series of exsiccatae into a single unit of which each set usually contains both elements, isotypes are present in numerous herbaria. The corresponding exsiccatae sheets with the admixed material of *Cicerbita polyclada* and *Lactuca denaensis* carry the printed label “Th. Kotschy, Pl. Pers. austr. Ed. R. F. Hohenacker. 1845 // 603. 662. *Lactuca polyclada* / Boiss. n. sp. // In glareosis alpis Kuh-Daëna. D. 14. Jul. 1842. / Pl. lactescens.” They are usually filed under *Lactuca* or *Cicerbita polyclada*, respectively, or, sometimes erroneously under *Cephalorrhynchus polycladus* (Boiss.) Kirp. The latter name is not a further homotypic synonym of *Lactuca polyclada*, but actually based on *Zollikoferia polyclada* Boiss., which represents a different mountainous species distributed from E Iran and Afghanistan to Central Asia, also with intricately and divaricately branched stems. Habitually, the latter species can readily be distinguished by its indurate and never inflated stems and branches; also it is not present in the Zagros mountains.

#### Distribution and habitat.

As far as we know, *Lactuca denaensis* is restricted to the higher elevations, probably above 3000 m, of Kuh e-Dena in the Zagros mountains of SW Iran (Fig. 4). From the rare material with subterranean parts preserved (e.g. on the sheets MO 6264530 and P 00750252 with isotypes), showing a subterranean caudex producing several cm long shoots vested with cataphylls below the leaf rosettes, it can be concluded that the species is a scree plant.

Additional specimen seen: Iran. Kogiluyeh & Boyer Ahmad: Dena [c. 30°56'N, 51°28'E], 3300 m, Sep 1955, Remaudière 5252E (IRAN 10625).

#### Etymology.

*Lactuca denaensis* is named after the Kuh e-Dena massif of the Zagros mountains, where the only two collections known to us come from.

#### Morphological affinities and delimitation.

**Figure 3.**
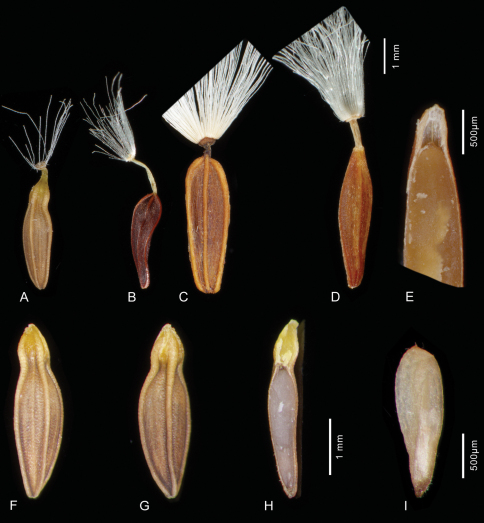
Achenes of the *Lactuca rosularis* group and *Cicerbita polyclada* – **A, F–I** *Lactuca hazaranensis*; complete achene (**A**); achene after loss of easily detachable beak, dorsal view (**F**); ventral view (**G**); longitudinal section of achene showing two segments, proximal large segment with embry, distal small segment solid with yellow tissue (**H**); embryo (**I**); **B** *Lactuca rosularis* **C** *Lactuca denaensis* **D–E** *Cicerbita polyclada*, complete achene (**D**) and longitudinal section of distal portion showing apical cavity (**E**). – **A–D** and **F–H** each to the same scale **A, F–I** from the holotype at IRAN **B** from Rechinger 55980 **B, C** from Kotschy 662 (B 100426936) **D–E** from Strauss 14091 (B 100312952)

Morphological comparison shows that *Lactuca denaensis* is most similar to *Lactuca rosularis*. Both share the rosulate habit with a woody caudex, glaucous leaves and small yellow-flowered capitula but also the compressed, beaked achenes (compare Fig. 3B and C), characterised by 4 (only exceptionally 5) similar and strongly prominent main ribs (two lateral and one median on either side, exceptionally dorsally 2) and a simple pappus without an outer series of minute hairs (see also Table 1).

#### Preliminary conservation status.

*Lactuca denaensis* is known only from the higher elevations of the Kuh e-Dena massif, which is part of a Protected Area, possibly also including the populations of the species. The species seems to be rare, since only two collections are known to us, but it has to be taken into account that it is rather inconspicuous. *Lactuca denaensis* must currently be assessed as Data Deficient (IUCN 2001) and an assessment of its populations in the field is strongly desirable.

### Lactuca hazaranensis

Djavadi & N. Kilian sp. nov.

urn:lsid:ipni.org:names:77118677-1

http://species-id.net/wiki/Lactuca_hazaranensis

Fig. 2 3A, F–I

#### Diagnosis. 

Habitually similar to *Lactuca rosularis* but clearly distinguished from it by the rosette leaves being undivided (instead of lyrately to irregularly pinnatifid to pinnatisect), the peduncles being distinct, c. 0.3–0.7(–1) cm long (instead of indistinct, 0–0.2 cm long), the achenes having a 0.3–0.5 mm (instead of (1.1–)3–5.3 mm) long beak and the achene body being transversally constricted and sectioned in the distal quarter into an embryo-containing proximal and a solid distal segment (instead of being unconstricted and unsectioned).

#### Holotype.

Iran. Kerman: Rayen, near Rayen falls [c. 29°33'N, 57°18'E], 2850 m, 10 Aug 2010, M. Eskandari & A. Torabi(IRAN 55199; photo at B).

#### Description. 

Perennial rosulate herb (Fig. 2A), with a taproot(?) and a woody caudex covered by the marcescent remains of old leaf bases. Stem one or a few per leaf rosette, erect, (2–)10–18 cm tall, branched already in lower half. Rosette leaves obovate to spatulate, (2–)5–11 × (1–)2–5 cm, somewhat glaucous; base semiamplexicaul, margin densely, coarsely and ± irregularly dentate-denticulate, apex subacute to acute. Lower and middle stem leaves spatulate to lanceolate, with auriculately clasping base, smaller, otherwise similar to rosette leaves; upper stem leaves distinctly smaller than lower and middle ones, lanceolate to ovate, with conspicuously auriculately clasping base, margin usually entire, apex acute to acuminate; uppermost stem leaves bractlike. Synflorescence (Fig. 2A) of a stem corymbosely paniculiform, with some to many capitula, axes wiry; peduncles c. 0.3–0.7(–1) cm long, capillaceous. Capitula with 7–14 flowers. Involucre (Fig. 2B–G) narrowly cylindric at anthesis, 6–7 mm long, not elongating during maturation; outer phyllaries imbricate, outermost ovate to narrowly ovate, 1.5–3 mm long, similar to the bracts on the peduncle, following ones gradually longer and ovate-lanceolate to lanceolate, the longest up to c. 1/2 as long as inner ones; inner phyllaries linear-lanceolate, 6–9, ± in one row, somewhat unequal in length, with ± narrow scarious margin. Receptacle flat to slightly convex, naked. Flowers with corolla yellow, ligule 5.5–6.5 mm long, tube shorter than ligule; anther tube without appendages 1.6–2 mm, basal appendages c. 0.4 mm, apical appendages 0.4 mm long; style arms 1.6–1.8 mm long. Achenes (Fig. 3A) homomorphic, including beak 3.2–3.6 mm long, corpus 2.9–3.2 mm long, up to 0.8–1.1 mm in diam., ellipsoidal, compressed, with a transversal constriction of 0.4–0.8 mm diam. in the distal 1/3–1/4, distal segment c. 1.5 × 1.5 mm, contracted into a stout, easily detachable beak c. 0.3–0.5 mm long; achene body (Fig. 3F–I) apart from the two lateral ribs with 1 similarly strong median rib on either side, rarely dorsally with 2 equally strong ribs, secondary ribs missing or rarely 1–2 per side; achene surface faintly transversally wrinkled, proximal segment brown, distal segment and beak yellowish, ribs straw-coloured to yellowish in distal segment; proximal segment containing the whitish embryo (Fig. 3H–I), distal segment containing yellowish tissue (Fig. 3H). Pappus simple, without an outer series of minute hairs, setae thin, white, 3–3.5 mm long, easily detachable. – Flowering and fruiting: June to August.

#### Distribution and habitat.

**Figure 4.**
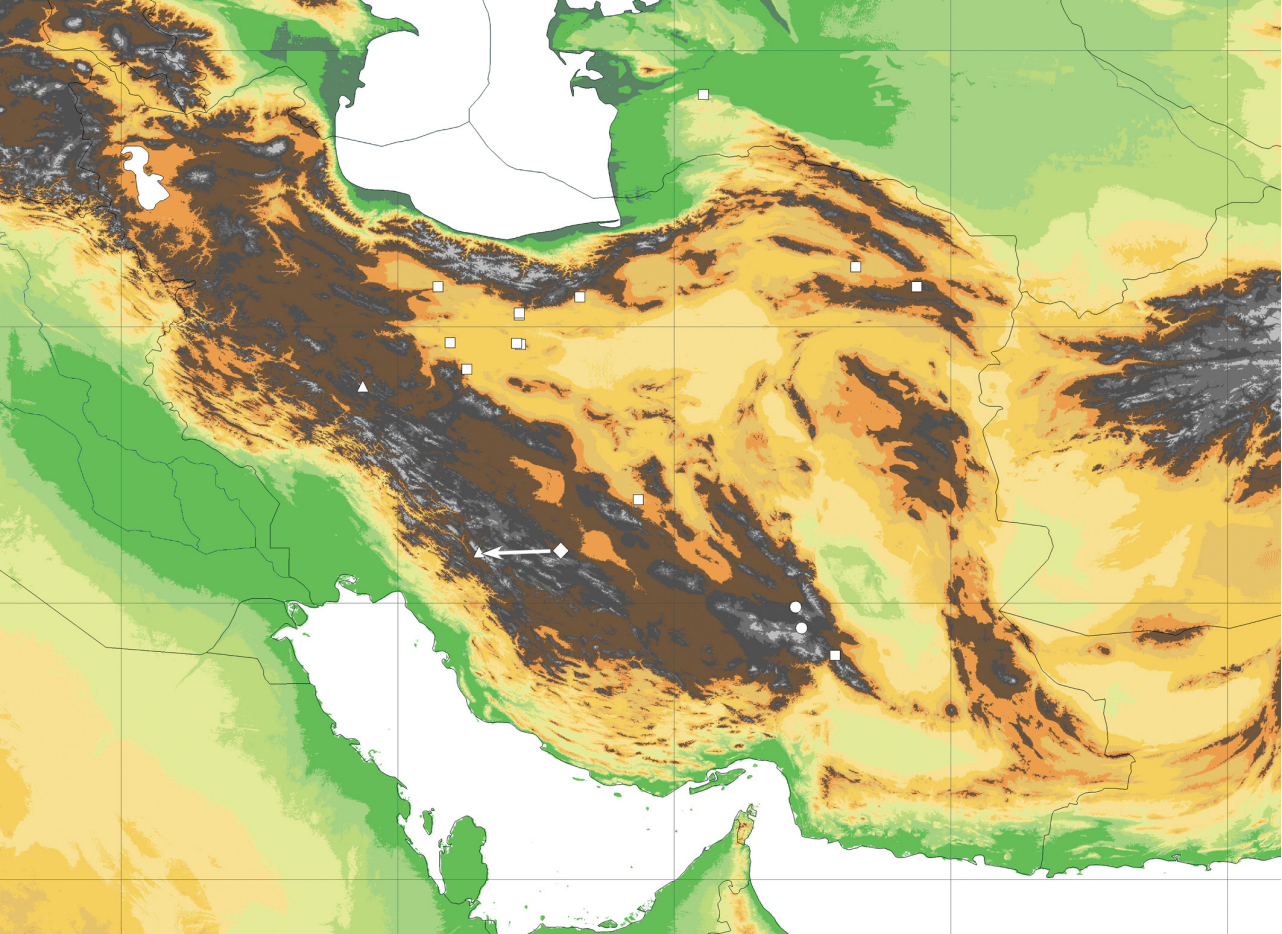
Distribution of *Lactuca hazaranensis* (circles) and the related species *Lactuca rosularis* (squares) and *Lactuca denaensis* (rhomb, actucal position as indicated by arrow), as well as of *Cicerbita polyclada* (triangles). – Georeferenced map based on the known collections (see Appendix and supplemented by collection cited in Kirpicznikov 1964 and Tuisl 1977) and generated with DIVA-GIS (Hijmans 2011) using an adaptation of the SRTM 90 m digital elevation data (CGIAR-CSI 2004).

The type collection of *Lactuca hazaranensis* comes from the northeastern foot of Mt Hezar, which rises to 4465 m elevation, and has been collected in the vicinity of the Rayen falls, at an altitude of 2850 m, in rock crevices. A second collection, with mature achenes of the precisely the same variant, was traced in the Berlin herbarium and had been made by J. Bornmüller in 1892 some 50 km further NW on rocks at an altitude of 3700 m on Mt Jupar (c. 29°55.8'N, 57°11.5'E; spelled “Khu-i-Dschupar” by Bornmüller, see also Freitag and Kuhle 1980), which is also situated in the Hazaran or Hezar-Lalezar mountain range (Fig. 4). Bornmüller (1939: 224), who determined this collection as *Lactuca rosularis*, characterised it as very rare on Mt Jupar, having only traced three tiny individuals (all preserved on the single sheet at B). The Hazaran or Hezar-Lalezar mountain range, which is mainly composed of limestone, is the highest mountain range in southeastern Iran and known as a local centre of endemism (Noroozi et al. 2010).

Additional specimen seen: Iran. Kerman: in rupibus summi jugii m. Kuh-i-Dschupar [Kuh-i-Jupar, c. 29°55.8'N, 57°11.5'E], 3700 m, 13 Jun 1892, J. Bornmüller 4119 (B).

#### Etymology.

*Lactuca hazaranensis* is named after its provenance, the Hazaran mountain massif in the Iranian province of Kerman, which is a southeastern outlier of the Zagros mountain range and reaches a maximum elevation of about 4500 m in the peak Kuh-e Hazar.

#### Morphological affinities and delimitation

. Morphological comparison revealed that the new species is most similar to both *Lactuca rosularis* and *Lactuca denaensis*. The three species are perennial rosette herbs of montane to high-montane environments, which are most likely closely related to each other, and considered here as the *Lactuca rosularis* group. They are all rare, being known from few collections only, and are endemic or almost so to the Iranian Highlands (Fig. 4). They share the rosulate habit with a woody caudex, the glaucous leaves, small yellow-flowered capitula and the principally same achene morphology. The differences between these three species are summarised in Table 1.

**Table 1.** Morphological differences between *Lactuca hazaranensis* and its relatives *Lactuca rosularis* and *Lactuca denaensis*, as well as between the latter and *Cicerbita polyclada*. Based on the material studied. – The investigated material is listed in the Appendix.

Features	*Lactuca hazaranensis*	*Lactuca rosularis*	*Lactuca denaensis*	*Cicerbita polyclada*
Rosette leaves: division	undivided	lyrately to irregularly pinnatifid to pinnatisect with large terminal lobe	undivided	undivided, towards base sometimes shallowly pinnately divided
Rosette leaves: margin	coarsely and ± irregularly dentate and denticulate	irregularly dentate and denticulate	± densely dentate and denticulate	subentire to dentate and denticulate
Branching of stem	with dominating main axis, corymbosely paniculiform	with dominating main axis, paniculiform to corymbosely paniculiform	subacaulescent,<br/> with dominating main stem only few cm long or branched from base, corymbosely paniculiform	± without dominating main axis, repeatedly divaricately branched from base
Branches	slender to capillaceous	± slender (to capillaceous)	slender to capillaceous	conspicuously inflated
Penduncles, length [cm], shape	c. 0.3–0.7(–1), capillaceous	0–0.2, capillaceous if developed	0.4–0.7, capillaceous	1–3.5, inflated if well developed
Cauline leaves	usually present, gradually reduced to bracts	basally present, soon reduced to bracts	usually absent, reduced to bracts	absent
Involucre, length [mm]	6–7	6–9(–10)	10–12	7–9
Corolla, colour	yellow	yellow	yellow	blue
Achene, length [mm]	3.2–3.6	(3.8–)6–8	4.8–5.7	3.4–5.2
Achene: corpus, length [mm]	2.9–3.2	2.2–3	4–5	3–4.2
Achene: ribs	4(–5), strongly prominent	4, strongly prominent	4(–5), strongly prominent	5, subprominent to subdistinct
Achene: beak, length [mm]	0.3–0.5	(1.1–)3–5.3	0.4–0.9(–2)	0.5–1
Achene: transversal constriction	present	absent	absent	absent
Pappus, length [mm]	3–3.5	2.5–3	5–6	3–4
Pappus: outer ring of minute hairs	absent	absent	absent	present

#### Preliminary conservation status.

*Lactuca hazaranensis* is known only from two localities c. 50 km apart, which are not in protected areas. The species seems to be rare, but it has to be taken into account that it is rather inconspicuous in its rocky environment. Members of the tribe are among the most favoured food of livestock, grazed wherever in reach and are therefore particularly threatened by overgrazing. *Lactuca hazaranensis* must currently be assessed as Data Deficient (IUCN 2001), but since the status Endangered seems not unlikely, an assessment of its populations in the field would be desirable.

#### Transversally constricted achenes of Lactuca hazaranensis aiding myrmecochory?

The shape of the achenes of *Lactuca hazaranensis* with the transversal constriction in the distal third (Fig. 3A, F–H) is curious. The fact that all achenes of all fruiting heads in two collections from different localities and centuries invariably show the same morphology, rules out the possibility that this achene variant represents a teratogenic manifestation.

Conspicuous transversal constrictions are, as far as we know, a very rare phenomenon in Asteraceae fruits. The present case is parallelled, however, by a few *Pulicaria* species of the Horn of Africa and southern Yemen (Wagenitz and Gamal-Eldin 1983 under *Sclerostephane*; Kilian 1999), which are likely not all closely related to each other (Englund et al. 2009) in contrast to what was thought initially. The possible function of these constrictions in the *Pulicaria* species is unknown.

In contrast to the cases in *Pulicaria*, where the constrictions chiefly affect the pericarp (Kilian 1999: fig. 2c, 3a, 4a, 5c), the constriction in *Lactuca hazaranensis* incompletely divides the achene into two segments (Fig. 3H). The large proximal segment contains the whitish embryo (Fig. 3I), the small distal segment is solid and of a yellowish tissue, which is partly identical partly contiguous with and therefore apparently derived from the intercostal yellowish pericarp tissue. The tissue of the embryo and the tissues of the distal segments are somewhat spatially separated from each other (Fig. 3H).

Conspicuous segmentation of the achene, although not precisely by a transversal constriction, is otherwise known from the probably unique case of the bispecific genus *Urospermum* (Cichorieae, Hypochaeridinae): the achenes of this genus consist of a proximal, compressed segment, which contains the embryo, and a larger, inflated distal segment tapering into the beak (for images, see *Urospermum picroides* in ICN 2011). In contrast to our case, in *Urospermum* both segments are separated from each other by a transversal wall and the distal segment is hollow (Lack and Leuenberger 1979).

A morphological transition towards a segmentation might perhaps be an achene with a cavity below the beak as it can be observed, e.g. in *Cicerbita polyclada* (Fig. 3D–E). Within subtribe Lactucinae, the achenes of *Cephalorrhynchus polycladus* (Boiss.) Kirp., not to be confused with the habitually similar *Cicerbita polyclada*, might represent an even stronger morphological transition (pers. com. A. Sennikov, Feb 2012); the presumably empty apical achene portion below the beak is, as stated by Kirpicznikov (1964: 351 + t. 20, fig. 8), somewhat narrower and separated by a very slight (non-waisted) constriction proximally.

Being shortly beaked and provided with a pappus, the achenes of *Lactuca hazaranensis* appear principally suited for wind dispersal (anemochory). The entire beak is, however, detachable at its base from the distal segment of the achene by slightest pressure and also the pappus setae are very easily detachable and are thus not functional for dispersal by wind. Finally, the solid distal segment brings additional weight and thus impedes wind dispersal.

Light microscopic histochemical analysis, using Sudan staining, of the yellow tissue of the distal segment of *Lactuca hazaranensis* revealed abundant presence of lipid drops, which were similarly found also in the embryo. Lipids are well known in the embryo of *Lactuca* as a major component of the reserves for the germination, accounting for 33 % dry weight of the achene in lettuce (*Lactuca sativa*) (Paulsen and Srivastava 1968; Srivastava and Paulsen 1968; Halmer et al. 1978), and for 35 % in the oilseed lettuce cultivar of *Lactuca sativa* with particularly large fruits grown in Egypt as a source for cooking oil (Křístková et al. 2008).

Their spatial separation from the embryo makes it unlikely that the lipid reserves of the distal segment are related to the germination. A potential function for the dispersal of the achene seems more probable, in particular in connection with the easy detachment of the achene beak. The detachment of the beak has not only a potentially atelechorous effect but the rupture also exposes the lipid reserves of the distal segment. The distal segment could thus perhaps be an “elaiosome”, a structure developing from seed or fruit tissue and aiding diaspore dispersal by ants (myrmecochory) in that it both attracts and rewards them (Bresinsky 1963; Lengyel et al. 2010). Usually, ants carry the diaspores into their nest, consume the lipid-rich tissue or feed it to their larvae and finally dump the diaspore in or outside their nest. Elaiosomes have convergently developed in seed plants many times, being known from 77 families and 334 genera (Lengyel et al. 2010). They are also long known from the Asteraceae (Sernander 1906; Nesom 1981), having been reported from many Cardueae species and also from five other tribes (Anthemideae, Arctotideae, Calenduleae, Heliantheae, Senecioneae), but not so far from the Cichorieae (Lengyel et al. 2010).

Usually, elaiosomes develop in Asteraceae at the base of the achenes from tissue separating the achene from the receptacle. In this way, the elaiosome is separated from the embryo by the indurate pericarp, which hinders the ants to get access to the embryo in the interior. The development of an elaiosome at the apex of the achenes and inside the pericarp appears in this context much less favourable. Apart from the presumably higher cost of this solution, the constriction only incompletely locks off the lipid reserves from the embryo, with the risk of its damage. In case the hypothesis of the myrmecochorous property of the distal achene segment of *Lactuca hazaranensis* is confirmed, e.g. by an experimental approach such as exemplified by Nesom (1981), it certainly would make an interesting case, considering both the chasmophytic growth of the plants and their apparent rarity.

The unparallelled and, so far as we know, transition-free occurrence of the transversally constricted achenes in the *Lactuca rosularis* group, is, independently of its potential function, a particularly striking evidence for a considerable developmental plasticity in achene features in the Lactucinae.

## Supplementary Material

XML Treatment for Lactuca denaensis

XML Treatment for Lactuca hazaranensis

## Appendix

**Specimens of related species investigated**

***Lactuca rosularis***

Iran. Khorasan: prope Robat-e-Safid inter Mashad et Torbat-e-Heydariyeh, [c. 35.72°N, 59.38°E], 1800–2000 m, 29 May 1977, Renz & Runemark in Rechinger 55980 (B). Semnan: Momen-abad [c. 35°32.5'N, 53°17.5'E], 1100–1150 m, 26 Jun 1992, Termeh, Moussavi & Tehrani (IRAN 54459). Tehran: 13 km E Eyvanki, [35°20.9'N, 52°12.3'E], 1100 m, 8 Jul 1974, Renz & Iranshahr, Rechinger 16608 (IRAN 10627). Tehran: 13 km W Garmsar [35°15.3'N, 52°11.8'E], 25 May 1984, Moussavi & Karavar (IRAN 10626). Qom: Qom to Qomrud, [c. 34°42.9'N, 50°57.3'E], 16 Oct 1972, Iranshahr & Moussavi (IRAN 10629). Kerman: Bam, Deh Bakri, 29.054°N, 57.913°E, 6 May 1969, Pazouki & Hashemi (IRAN 10628).

***Cicerbita polyclada***

Iran. Kogiluyeh & Boyer Ahmad: [in glareosis alpis] Kuh-Daena [c. 30°56'N, 51°28'E], [Jul 1842], [Kotschy 603 ed. R. F.] Hohenacker (B 100312954 isolectotype). Markazi: Kuh-Shasinde [nr. Shahsand, c. 33°56'N, 49°22'E], Conglomeratabhänge, 18 Jul 1902, T. Strauss 14091 (B 100312952, 100312953).
